# Association of cigarette smoking intensity and pack-years with metabolic syndrome: A cross-sectional study

**DOI:** 10.6026/973206300220966

**Published:** 2026-02-28

**Authors:** Nishath Sayed Abdul, Shivakumar Ganiga Channaiah, Shubham Patel

**Affiliations:** 1 Department of Oral Pathology, People's College of Dental Sciences and Research Centre, People's University, Bhopal, Madhya Pradesh, India; 2 Department of Oral Medicine and Radiology, People's College of Dental Sciences and Research Centre, Peoples University, Bhopal, Madhya Pradesh, India; 3 Department of Oral Pathology and Microbiology, People's College of Dental Sciences and Research Centre, Peoples University, Bhopal, Madhya Pradesh, India

**Keywords:** Cigarette smoking, pack-years, smoking intensity, metabolic syndrome, cardiometabolic risk, cross-sectional study

## Abstract

The relationship between cigarette smoking intensity, cumulative exposure and metabolic syndrome remains inadequately characterized despite smoking being a known metabolic risk factor. Therefore, it is of interest to examine the association of smoking status, intensity and pack-years with metabolic syndrome and its individual components among 200 adults using standardized questionnaires and logistic regression analysis. Metabolic syndrome was present in 35.0% of participants and showed a progressive increase from never smokers (24.4%) to former smokers (35.0%) and current smokers (48.6%). Among current smokers, prevalence rose in a dose-dependent manner from light to heavy smoking and was accompanied by higher frequencies of central obesity, low HDL cholesterol and elevated triglycerides. Both smoking intensity and cumulative pack-year exposure were independently associated with a graded increase in the prevalence and odds of metabolic syndrome. By showing a distinct dose-dependent relationship between cigarette smoking intensity, cumulative pack-years, and metabolic syndrome instead of evaluating smoking status alone, the current study builds on previous research. In addition to highlighting cumulative smoking exposure as an independent and clinically significant predictor of metabolic syndrome, this offers more support for quantitative risk stratification.

## Background:

Metabolic syndrome (MetS) is the cluster of inter-related cardiometabolic abnormalities-central obesity, dysglycaemia, dyslipidaemia and elevated blood pressure-that substantially raises the risk of atherosclerotic cardiovascular disease, type 2 diabetes and several end-organ complications [1]. In patients with chronic liver disease, metabolic syndrome also seems to increase the risk of hepatocellular carcinoma, underscoring its systemic effects beyond classic cardiovascular outcomes [2]. Because many of these components are modifiable, elucidation of upstream behavioural determinants remains a priority for prevention strategies [3]. Cigarette smoking is a major modifiable risk factor that affects multiple pathways associated with metabolic syndrome. Cohort and life-course studies have demonstrated that the prevalence and incidence of metabolic syndrome are higher among current and long-term smokers, revealing dose- response relationships between cumulative smoking exposure and metabolic risk [4-5]. A similar clustering of metabolic abnormalities has been found among users of other combusted tobacco products, such as waterpipes, suggesting that tobacco-related toxic burden, rather than product type, is paramount [6]. In some high-risk groups, including hypertensive postmenopausal women, smoking in the context of metabolic syndrome has been associated with increased carotid intima-media thickness and impaired endothelial function, suggesting a deleterious interaction between tobacco exposures and pre-existing metabolic dysfunction [7]. Previous studies suggest that smoking often clusters with other harmful behaviours and risk factors that exacerbate the risk profiles for coronary heart disease and diabetes [8,9-10].

Neuroimaging provides evidence that characteristic changes in brain metabolism accompany cigarette craving and dependence, which can facilitate the maintenance of high-intensity smoking in susceptible individuals [9]. From a systemic viewpoint, cumulative smoking quantified as pack-years has been associated with chronic inflammatory and dermatological conditions, thereby justifying its use as an integrative marker of long-term toxic exposure [8]. In turn, physical activity and daily ambulatory patterns are similarly crucial modifiers of cardiometabolic risk: higher activity is related to better outcomes in smoking-related disorders, while step-count and intensity profiles differently relate to metabolic syndrome [6, 7]. These findings emphasize the importance of taking into account both smoking habits per se and concurrent lifestyle attributes in the study of metabolic clustering [6, 7, 10]. Nevertheless, most epidemiological studies still categorize smoking in broad categories (never, former, current) and do not make any differential classification of current intensity of smoking and the accumulation of exposure in a way that would enable an independent analysis of their associations with metabolic syndrome [2,3- 4,8,11]. Against this background, the present cross-sectional study was designed to evaluate, in an adult population, how cigarette smoking intensity and cumulative pack-year exposure are associated with the presence of metabolic syndrome and its components, while accounting for key demographic and lifestyle covariates, so as to refine the understanding of smoking-related metabolic risk in addition to established guideline-driven prevention frameworks. Therefore, it is of interest to evaluate the associations of cigarette smoking intensity and cumulative pack-year exposure with metabolic syndrome and its individual components in adults.

## Materials and Methods:

## Study protocol:

This cross-sectional analytical study was conducted in adult outpatient clinics of a tertiary care teaching hospital. The source population comprised adults attending for routine consultation and opportunistic screening in general medicine and lifestyle clinics. Data collection was carried out over a period of 12 months using a standardised, piloted protocol.

## Participants and eligibility criteria:

Inclusion criteria included men and women aged 18-70 years of age who were permanent residents in the hospital catchment area and provided written informed consent. They had to be able to communicate in the local language or English and provide a reliable history of smoking and medical conditions. The exclusion criteria included acute illness requiring emergency care, decompensated heart failure, chronic kidney disease on dialysis, pregnancy, known malignancy, major psychiatric disorders interfering with recall and systemic corticosteroid or weight-loss pharmacotherapy. Those with missing key data on smoking, anthropometry, blood pressure, fasting glucose, or lipid profile were excluded from the final analysis.

## Recruitment and sampling:

The minimum required sample size was therefore approximately 200 participants and this number of consecutive eligible adults was recruited.

The sample size for this cross-sectional study was estimated using the single-proportion formula: (see PDF)

where n is the required sample size, Z_(1-α/2)_ is the standard normal deviate for a 95% confidence level (1.96), pis the expected prevalence of metabolic syndrome and dis the allowable absolute error. A prevalence of 25% (p = 0.25) was assumed from prior regional data and the precision was set at 6 percentage points (d = 0.06).

Substitution gave:

n = (1.96)^2^ x 0.25 x 0.75 / (0.06)^2^ ≈ 200

## Assessment of cigarette smoking:

Data were collected about exposure to cigarette smoking through a structured interviewer-administered questionnaire. Participants' status was categorized as never, former, or current smokers according to the lifetime and current use of at least 100 cigarettes. Information on age at initiation, total years of smoking, average number of cigarettes per day and age at cessation (for former smokers) was collected among ever-smokers.

Cumulative exposure in pack-years was calculated using the formula:

Pack-years = number of cigarettes per day / 20 x years of smoking

Current smoking intensity was defined as the average number of cigarettes smoked per day in the past 12 months and categorised as light (<10 cigarettes/day), moderate (10-19 cigarettes/day), or heavy (≥20 cigarettes/day).

## Assessment of covariates:

Assessment of socio-demographic variables included age, gender, education, occupation and marital status. The lifestyle factors assessed included alcohol use, dietary habits and physical activity. Alcohol intake was recorded as frequency and typical quantity and categorized as non-drinker, occasional, or regular drinker. Physical activity was assessed using a brief validated tool and classified as low, moderate, or high. Past history of hypertension, diabetes, dyslipidaemia and cardiovascular disease and family history of cardiometabolic conditions were obtained from self-report and, when possible, verified against medical records.

## Anthropometric and blood pressure measurements:

All the measurements were carried out by trained staff using calibrated instruments. Body weight was measured to the nearest 0.1 kg with a digital scale and height to the nearest 0.1 cm with a stadiometer; body mass index was calculated as kg/m^2^. Waist circumference was measured at the midpoint between the lower rib and iliac crest at the end of expiration using a non-stretch tape; two readings were taken and the mean calculated. Blood pressure was measured in a seated position after at least 5 minutes of rest using an automated validated device; two measurements 2 minutes apart were obtained and averaged, with a third reading if values differed markedly.

## Definition of metabolic syndrome:

Metabolic syndrome was defined by harmonised criteria requiring at least three of the following: (1) increased waist circumference according to ethnicity-specific cut-offs; (2) elevated fasting plasma glucose or diagnosed diabetes/antidiabetic treatment; (3) raised triglycerides or lipid-lowering therapy specific for hypertriglyceridemia; (4) reduced HDL cholesterol or treatment for low HDL; and (5) elevated blood pressure or current antihypertensive therapy [1]. Definitions of BMI, physical activity, alcohol use, smoking status and smoking intensity and pack-year categories were as outlined above.

## Statistical analysis protocol:

Continuous variables were presented as mean ± standard deviation or median (interquartile range) and categorical variables as counts and percentages. We compared the baseline characteristics across smoking and pack-year categories by using chi-square tests for categorical variables and one-way ANOVA or Kruskal-Wallis tests for continuous variables. We assessed the association of smoking intensity and pack-years with metabolic syndrome by logistic regression. Initial univariable models were followed by multivariable models adjusted a priori for age, sex, BMI, physical activity, alcohol use and relevant clinical covariates. Results were presented as odds ratios (ORs) and 95% CIs. We assessed the linear trend across ordered smoking and pack-year categories by modelling the category medians as continuous variables. A two-sided p value <0.05 was regarded as statistically significant.

## Results:

**Figure 1:**
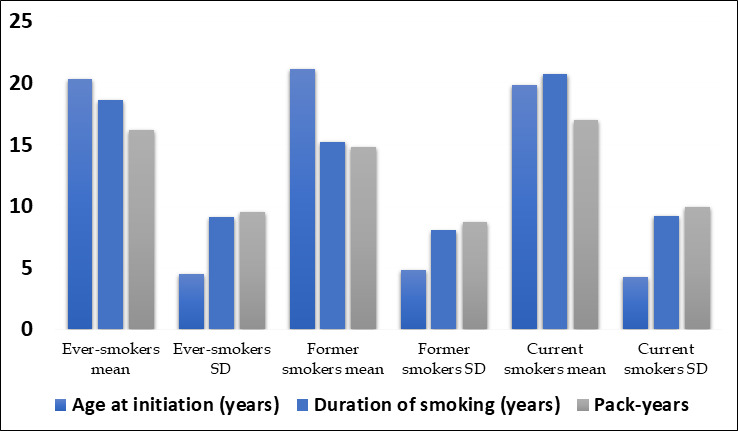
Continuous smoking characteristics among ever-smokers (n = 110)

**Figure 2:**
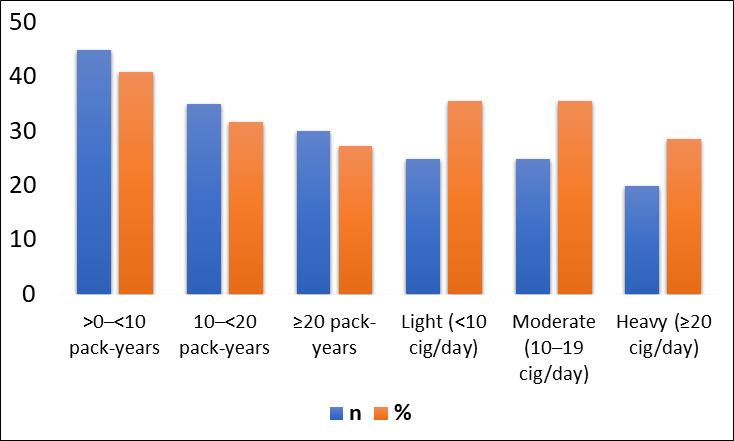
Categorical smoking characteristics among ever-smokers (n = 110)

**Figure 3:**
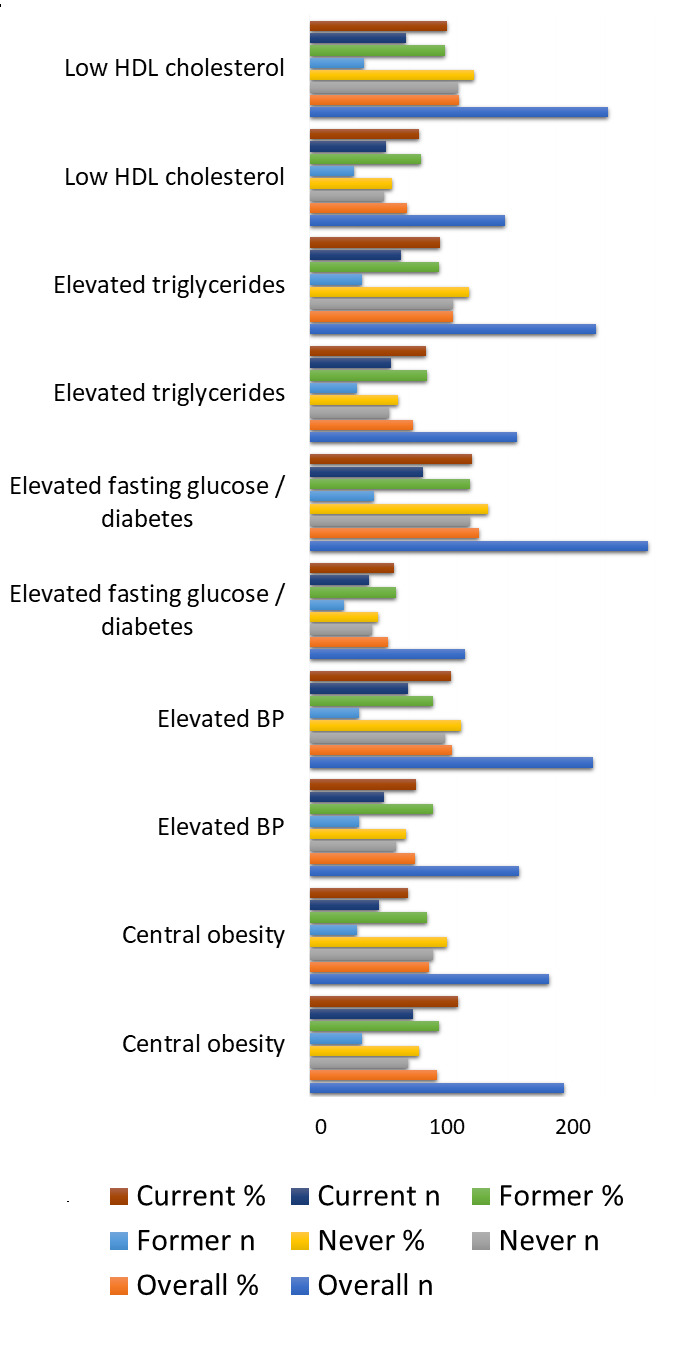
Prevalence of metabolic syndrome components by smoking status

On average, former and current smokers were slightly older than never smokers; both groups also had marginally higher BMI and waist circumference, although mean values stayed in the overweight range across all groups (Table 1). There were small absolute differences in blood pressure and lipid parameters, with current smokers showing slightly higher mean systolic blood pressure, triglycerides and LDL cholesterol and lower HDL cholesterol compared with never smokers. The proportion of men was higher among former and current smokers than among never smokers and current smokers more often reported regular alcohol consumption and low physical activity, whereas never smokers more often reported no alcohol use and higher activity levels. Crude prevalence of metabolic syndrome was lowest in never smokers (24.4%), intermediate in former smokers (35.0%), and highest in current smokers (48.6%) (Table 1). The average age at the start of smoking was in the early twenties, with current smokers starting slightly earlier than former smokers (Figure 1). Current smokers also had a longer average duration of smoking and higher average pack- years. In categorizing cumulative exposure, around 41% of ever-smokers fell into the >0-<10 pack-year group, about 32% into the 10-<20 pack-year group and 27% into the ≥20 pack-year group. In current smokers, light, moderate and heavy categories of intensity were similarly represented, indicating a spread of current daily cigarette consumption (Figure 2). Individual metabolic syndrome components also demonstrated variation by smoking status (Figure 3). Central obesity and low HDL cholesterol were more common among current smokers compared with never smokers, while elevated triglycerides and elevated fasting glucose/diabetes showed higher proportions among former and current smokers than among never smokers. Taken together, more than half of current smokers met criteria for central obesity and approximately 44-47% had low HDL cholesterol or elevated triglycerides, whereas never smokers demonstrated lower proportions for each component. These patterns are consistent with the higher crude prevalence of metabolic syndrome observed in the current-smoker group.

**Figure 4:**
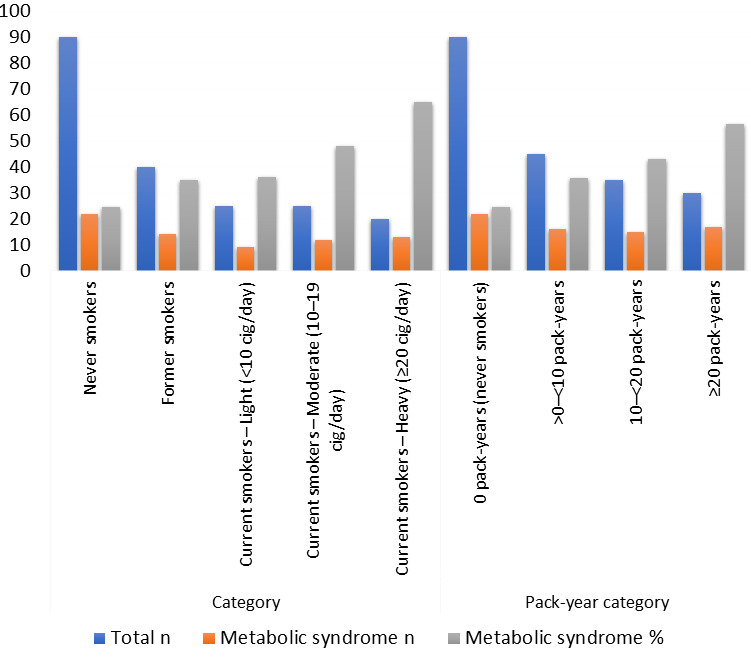
Prevalence of metabolic syndrome by smoking status and current smoking intensity and by cumulative smoking exposure (pack-years)

Examining metabolic syndrome across smoking status and current smoking intensity revealed a graded pattern (Figure 4). The prevalence of metabolic syndrome increased from 24.4% in never smokers to 35.0% in former smokers and then continued to rise across light, moderate and heavy current smoking, peaking at 65.0% in heavy smokers (≥20 cigarettes/day). A similar gradient was observed when cumulative exposure was expressed as pack-years. Participants with 0 pack-years had a metabolic syndrome prevalence of 24.4%, which increased to 35.6% in the >0-<10 pack-year group, 42.9% in the 10-<20 pack-year group and 56.7% in the ≥20 pack-year group. Based on these findings, both current intensity and cumulative smoking exposure appeared to have dose-related associations with metabolic syndrome. In univariable models using never smokers as the reference (Table 2), former smokers had higher but not statistically significant odds of metabolic syndrome (OR 1.66, 95% CI 0.74-3.73). Among current smokers, light smokers had a similar non-significant elevation in odds (OR 1.74, 95% CI 0.67-4.49), whereas moderate and heavy smokers had progressively higher and statistically significant odds of metabolic syndrome (OR 2.85, 95% CI 1.14-7.16 and OR 5.74, 95% CI 2.03-16.19, respectively) (Table 2). When smoking was modeled by pack-year categories, the odds of metabolic syndrome increased with higher exposure: compared with 0 pack-years, ORs were 1.71 (95% CI 0.78-3.71) for >0-<10 pack-years, 2.32 (95% CI 1.02-5.29) for 10-<20 pack-years and 4.04 (95% CI 1.70-9.62) for ≥20 pack-years (Table 3).

## Discussion:

The findings of this study have a number of important implications for risk assessment and the development of prevention strategies in daily clinical practice. The clear dose-response relationship between the intensity of smoking, pack-years and metabolic syndrome points to the fact that the metabolic effects of smoking are not limited to a simple yes/no pattern of exposure but depend on both current consumption and cumulative burden. These facts support the systematic introduction of quantitative measures of smoking, especially pack- years and current intensity categories, into cardiometabolic risk profiling rather than relying on binary or coarse smoking status categories. Also, the finding of markedly higher odds of metabolic syndrome among heavy current smokers and subjects with greater pack- year exposure suggests that these subgroups may represent priority targets for intensified screening of metabolic abnormalities and structured smoking-cessation interventions within primary care and ambulatory settings. The association of cigarette smoking exposure with metabolic syndrome had been placed in a broader context of clustered lifestyle risk factors. Lifetime alcohol drinking patterns were earlier found to be associated with the prevalence of metabolic syndrome and suggested that cumulative behavioural risk exposure, rather than single-point measures, was central to cardiometabolic clustering [12, 13]. In this context, smoking intensity and duration were not isolated exposures but were embedded in patterns of drinking, physical inactivity and sociodemographic context, all of which shaped the metabolic phenotype. Epidemiological evidence consistently showed that active cigarette smoking was related to a higher prevalence of metabolic syndrome in various populations [14, 15,16-17]. In the Korea National Health and Nutrition Examination Survey, current smoking was positively associated with metabolic syndrome after adjustment for multiple covariates [14]. Similar relationships were seen among middle-aged Japanese male office workers, in whom smokers had greater risk of metabolic syndrome compared with non-smokers [15]. In Japanese community samples, smoking were not only associated with metabolic syndrome but also with carotid arteriosclerosis, with the important implication that metabolic clustering in smokers was paralleled by structural vascular changes [16]. Another study that assessed smoking in conjunction with alcohol use, exercise, education and family history demonstrated that smoking was independently associated with metabolic syndrome as defined by the ATP III criteria, even when other lifestyle factors were considered [17]. Taken together, these findings suggested that the intensity and cumulative exposure to smoking needed explicit consideration when metabolic syndrome was assessed. The persistence of metabolic risk after smoking cessation further underlined the importance of cumulative dose. In a longitudinal analysis, the risk of metabolic syndrome remained elevated even 20 years after cessation, which suggested that prior exposure had imprinted a long-term metabolic legacy [18]. Cross-sectional studies from Taiwan and Puerto Rico reported that both current and former smokers have higher odds of metabolic syndrome than never-smoking counterparts and that individual components such as abdominal obesity, dyslipidaemia and impaired fasting glucose all contributed to this excess [19, 20]. These findings implicated pack-years and duration of smoking, rather than current intensity perse, as critical determinants of metabolic syndrome risk. Several studies specifically addressed smoking intensity and dose-response patterns. Among an Italian sample, metabolic syndrome prevalence differed between light and heavy smokers, with the highest rates among heavy smokers, suggesting a quantitative gradient of risk [21]. A cross-sectional study from Maracaibo, Venezuela, demonstrated that smoking was associated with several components of metabolic syndrome, including elevated triglycerides, low HDL cholesterol and raised blood pressure, in further support of a clustering of adverse metabolic traits in smokers [22]. More recent work from Korea reported interactive associations between smoking and physical activity, wherein high physical activity partly mitigated the adverse metabolic impact of smoking, although smokers still showed higher metabolic syndrome risk than did non- smokers [23]. These findings indicated that the intensity of smoking and pack-years interacted with other behaviours in shaping the metabolic profile.

Gender and physical activity seemed to modify the association between smoking and metabolic syndrome in current smokers. One cross- sectional study from Taiwan showed that the association of smoking with metabolic syndrome varied by sex and physical activity level; male smokers with low activity had the highest risk, while physically active smokers had comparatively lower, though still elevated, risk [24]. Another study reported that heavy smokers had worse metabolic syndrome outcomes and higher anxiety levels compared to light smokers, suggesting that psychological burden and smoking-related stress may accompany or amplify metabolic disturbances [25]. These observations supported a multidimensional view wherein intensity and cumulative exposure were interlinked with sex, psychosocial factors and activity patterns. Mechanistic studies provided further context for interpreting the association between smoking dose and metabolic syndrome. Smoking was found to affect body weight, body fat distribution and insulin resistance in a complex manner, with evidence of lower overall body weight but greater central adiposity and higher insulin resistance indices in smokers [26]. Meta-analytic evidence from prospective cohorts demonstrated that active smoking was associated with an increased risk of incident metabolic syndrome, reinforcing that the observed associations were not merely cross-sectional artefacts [27]. Experimental work further suggested that even brief smoking exposure increased insulin resistance, indicating that metabolic disturbances could occur relatively early during exposure before accumulating with greater intensity and duration [28]. These findings supported biological plausibility for a dose-related association between smoking and metabolic syndrome. Alcohol consumption and its interaction with smoking, constituted an additional layer of complexity. Alcohol intake in Korean adults had a U- or J-shaped association with metabolic syndrome, with high consumption and certain patterns of drinking associated with higher prevalence [29]. In conjunction with smoking, this suggested that combined exposure to tobacco and alcohol may have additive or synergistic effects on metabolic clustering and that any analysis of smoking intensity and pack-years needed to consider drinking patterns [13, 17 and 29]. More broadly, metabolic syndrome itself was recognised as an important intermediate endpoint in the pathway to cardiovascular disease and type 2 diabetes, through multiple mechanistic pathways involving visceral adiposity, dyslipidaemia, low-grade inflammation and endothelial dysfunction [30]. Clarification of how smoking dose and duration modulates this cluster was thus clinically relevant beyond the syndrome definition alone. At the lipid level, smoking was associated not only with conventional abnormalities but also with changes in lipoprotein particle size. Indeed, evidence documented that smokers tended to have smaller, denser LDL and HDL particles, more atherogenic and less protective, respectively [31]. The association of smoking exposure with metabolic syndrome in our study was generally consistent with previous reports. Current smokers in this sample had higher rates of metabolic syndrome than never smokers, as in numerous large cross-sectional and cohort studies and risks rose progressively with dose and duration of smoking, as reflected in life-course and dose-response findings from Korean, Japanese, Taiwanese and other populations [2, 3, 14, 15-16, 19, 20, 21-22 and 27]. The graded pattern across light, moderate and heavy smoking and across rising pack-year categories was closely consistent with reports that heavier or long-term smokers bear the greatest metabolic burden [3, 14,18,19, 21, 22 and 27]. At the component level, the higher frequencies of central obesity, low HDL cholesterol and elevated triglycerides among current smokers in the present study were consistent with reports that smoking clustered with abdominal adiposity and an atherogenic lipid profile and with evidence indicating adverse effects on lipoprotein particle characteristics and insulin resistance [16, 19, 22, 26 and 31]. The intermediate metabolic syndrome prevalence in former smokers in this study, falling between never and current smokers, was also consistent with data suggesting that some excess risk remained years after cessation and that cumulative exposure continued to be relevant even after quitting [2, 18]. Our findings, therefore, are according to the broader literature linking smoking, metabolic syndrome and downstream vascular and hepatic risk, indicating that tobacco exposure, together with clustered metabolic abnormalities, contributed to vascular damage and cardiometabolic events [5, 10, 11, 26 and 31]. Unlike studies that formally explored effect modification by physical activity, sex, or psychological factors [7, 10, 23, 24-25 and 29], the current study adjusted for major lifestyle variables but did not evaluate interactions; however, the direction and magnitude of the main associations were concordant with those more detailed models [2, 3, 14,15- 16, 19,20, 21, 22-23, 27].

## Limitations:

A few limitations must be taken into account in the interpretation of the findings of this study. Its cross-sectional design allowed no inference about temporality or causality between smoking exposure and metabolic syndrome. The sample was from one tertiary-care outpatient clinic and might not have been fully representative of the general population, reducing external generalisability. Smoking history was self-reported without biochemical validation and, especially for duration and intensity, may have been subjected to recall or social desirability bias. Residual confounding due to unmeasured or incompletely measured covariates, including detailed dietary patterns, psychosocial factors, or occupational exposures, cannot be excluded despite adjustment for major covariates. The sample size, while sufficient for the primary analyses, restricted precision for certain subgroup estimates, especially at extremes of smoking exposure.

## Conclusion:

In this cross-sectional analysis of ambulatory adults, higher cigarette smoking intensity and greater cumulative pack-year exposure were associated with an increased likelihood of metabolic syndrome and adverse metabolic profiles. A clear graded relationship was observed across smoking intensity and cumulative exposure categories, indicating that quantitative smoking measures provide important information on metabolic clustering. These findings support incorporating detailed smoking history into cardiometabolic risk assessment, with heavier and long-term smokers requiring closer surveillance and targeted preventive strategies.

## Figures and Tables

**Table 1:** Demographic data

**Characteristic**	**Overall (n = 200)**	**Never smokers (n = 90)**	**Former smokers (n = 40)**	**Current smokers (n = 70)**
Age, years, mean ± SD	48.7 ± 11.5	46.2 ± 11.2	52.8 ± 9.8	49.5 ± 12.0
Male sex, n (%)	120 (60.0)	40 (44.4)	28 (70.0)	52 (74.3)
BMI, kg/m^2^, mean ± SD	26.7 ± 4.1	26.1 ± 3.9	27.4 ± 4.1	27.0 ± 4.2
Waist circumference, cm, mean ± SD	92.8 ± 10.7	90.2 ± 10.8	94.1 ± 9.7	95.3 ± 10.5
Systolic BP, mmHg, mean ± SD	128.9 ± 16.1	126.0 ± 15.2	130.4 ± 16.3	132.1 ± 16.8
Diastolic BP, mmHg, mean ± SD	80.2 ± 9.1	79.3 ± 8.8	80.0 ± 9.0	81.0 ± 9.5
Hypertension, n (%)	71 (35.5)	30 (33.3)	17 (42.5)	24 (34.3)
Diabetes mellitus, n (%)	45 (22.5)	20 (22.2)	10 (25.0)	15 (21.4)
Dyslipidaemia, n (%)	67 (33.5)	28 (31.1)	15 (37.5)	24 (34.3)
Fasting plasma glucose, mg/dL, mean ± SD	104 ± 20	100 ± 18	106 ± 20	107 ± 22
Triglycerides, mg/dL, mean ± SD	160 ± 55	150 ± 50	160 ± 55	170 ± 60
HDL cholesterol, mg/dL, mean ± SD	45 ± 11	47 ± 12	44 ± 11	43 ± 10
LDL cholesterol, mg/dL, mean ± SD	121 ± 35	118 ± 34	122 ± 36	125 ± 37
Physical activity - Low, n (%)	69 (34.5)	25 (27.8)	14 (35.0)	30 (42.9)
Physical activity - Moderate, n (%)	88 (44.0)	40 (44.4)	18 (45.0)	30 (42.9)
Physical activity - High, n (%)	43 (21.5)	25 (27.8)	8 (20.0)	10 (14.3)
Alcohol - Non-drinker, n (%)	107 (53.5)	60 (66.7)	22 (55.0)	25 (35.7)
Alcohol - Occasional drinker, n (%)	55 (27.5)	25 (27.8)	10 (25.0)	20 (28.6)
Alcohol - Regular drinker, n (%)	38 (19.0)	5 (5.6)	8 (20.0)	25 (35.7)
Metabolic syndrome present, n (%)	70 (35.0)	22 (24.4)	14 (35.0)	34 (48.6)

**Table 2:** Association of smoking status and intensity with metabolic syndrome (univariable logistic regression)

**Smoking category**	**Metabolic syndrome, n/N (%)**	**Odds ratio vs never (95% CI)**	**p-value**
Never smokers	22/90 (24.4)	1.00 (reference)	-
Former smokers	14/40 (35.0)	1.66 (0.74-3.73)	0.217
Current smokers - Light (<10 cig/day)	9/25 (36.0)	1.74 (0.67-4.49)	0.253
Current smokers - Moderate (10-19 cig/day)	12/25 (48.0)	2.85 (1.14-7.16)	0.026
Current smokers - Heavy (≥20 cig/day)	13/20 (65.0)	5.74 (2.03-16.19)	0.001

**Table 3:** Association of pack-year categories with metabolic syndrome (univariable logistic regression)

**Pack-year category**	**Metabolic syndrome, n/N (%)**	**Odds ratio vs 0 pack-years (95% CI)**	**p-value**
0 pack-years (never smokers)	22/90 (24.4)	1.00 (reference)	-
>0-<10 pack-years	16/45 (35.6)	1.71 (0.78-3.71)	0.178
10-<20 pack-years	15/35 (42.9)	2.32 (1.02-5.29)	0.046
≥20 pack-years	17/30 (56.7)	4.04 (1.70-9.62)	0.002
